# Optically pumped magnetometers detect altered maximal muscle activity in neuromuscular disease

**DOI:** 10.3389/fnins.2022.1010242

**Published:** 2022-11-29

**Authors:** Lorenzo Semeia, Thomas Middelmann, Sangyeob Baek, Davide Sometti, Hui Chen, Alexander Grimm, Holger Lerche, Pascal Martin, Cornelius Kronlage, Christoph Braun, Philip Broser, Markus Siegel, Maria-Sophie Breu, Justus Marquetand

**Affiliations:** ^1^Graduate Training Centre of Neuroscience, International Max Planck Research School, University of Tübingen, Tübingen, Germany; ^2^Department of Neural Dynamics and Magnetoencephalography, Hertie Institute for Clinical Brain Research, University of Tübingen, Tübingen, Germany; ^3^MEG Center, University of Tübingen, Tübingen, Germany; ^4^Department of Biosignals, Physikalisch-Technische Bundesanstalt (PTB), Berlin, Germany; ^5^CIMeC, Center for Mind/Brain Sciences, University of Trento, Rovereto, Italy; ^6^Department of Neurology and Epileptology, Hertie Institute for Clinical Brain Research, University of Tübingen, Tübingen, Germany; ^7^Department of Psychology and Cognitive Science, University of Trento, Rovereto, Italy; ^8^Children’s Hospital of Eastern Switzerland, St. Gallen, Switzerland; ^9^Center for Integrative Neuroscience, University of Tübingen, Tübingen, Germany

**Keywords:** optically pumped magnetometers (OPM), neuromuscular disease (NMD), electromyogram (EMG), Charcot-Marie-Tooth, ATTR amyloidosis, muscle activity, myotonia congenita, magnetomyography (MMG)

## Abstract

Optically pumped magnetometers (OPM) are quantum sensors that enable the contactless, non-invasive measurement of biomagnetic muscle signals, i.e., magnetomyography (MMG). Due to the contactless recording, OPM-MMG might be preferable to standard electromyography (EMG) for patients with neuromuscular diseases, particularly when repetitive recordings for diagnostic and therapeutic monitoring are mandatory. OPM-MMG studies have focused on recording physiological muscle activity in healthy individuals, whereas research on neuromuscular patients with pathological altered muscle activity is non-existent. Here, we report a proof-of-principle study on the application of OPM-MMG in patients with neuromuscular diseases. Specifically, we compare the muscular activity during maximal isometric contraction of the left rectus femoris muscle in three neuromuscular patients with severe (Transthyretin Amyloidosis in combination with Pompe’s disease), mild (Charcot-Marie-Tooth disease, type 2), and without neurogenic, but myogenic, damage (Myotonia Congenita). Seven healthy young participants served as the control group. As expected, and confirmed by using simultaneous surface electromyography (sEMG), a time-series analysis revealed a dispersed interference pattern during maximal contraction with high amplitudes. Furthermore, both patients with neurogenic damage (ATTR and CMT2) showed a reduced variability of the MMG signal, quantified as the signal standard deviation of the main component of the frequency spectrum, highlighting the reduced possibility of motor unit recruitment due to the loss of motor neurons. Our results show that recording pathologically altered voluntary muscle activity with OPM-MMG is possible, paving the way for the potential use of OPM-MMG in larger studies to explore the potential benefits in clinical neurophysiology.

## Introduction

The hallmark of most neuromuscular diseases is muscle weakness, which often emerges because of motor neuron loss (Garg et al., 2017). To compensate for this motor neuron loss, collateral sprouting from neighboring motor neurons arises and leads to a re-innervation of muscle fibers, ultimately increasing the size of individual motor units (Edds, 1953; Kugelberg et al., 1970; Milner-Brown et al., 1974; Zochodne, 2012). This compensatory mechanism leads to several electrophysiologically measurable alterations, for example, during maximal voluntary activation of a muscle. In healthy individuals, during maximal voluntary activation, a dense interference signal is detected because of the activation of a large number of motor units that vary in size and frequency. In neurogenic damage, this interference signal is dispersed because fewer motor units can contribute to the interference. At the same time, the interference signal also shows a high amplitude, since the remaining compensatory oversized motor units innervate a larger number of muscle fibers (i.e., a larger signal source) (Willison, 1964; Nandedkar et al., 1986a,b). These measurable changes are used in everyday clinical routines to detect not only a single neurogenic damage but also to establish a diagnosis of various neuromuscular diseases, such as motor polyneuropathy (Dumitru et al., 2002; Mills, 2005; Preston and Shapiro, 2012). Electromyography (EMG) is the modality of choice for detecting these pathological electrophysiological changes, either using electrodes placed on the skin surface (sEMG) or—as a gold standard—needle electrodes penetrating the muscle (nEMG) (Lipa and Han, 2012; Morrison, 2016; Stålberg et al., 2019). Even though nEMG is well-established, has a high signal-to-noise ratio and a narrow and therefore precise detection area, this modality is often perceived as unpleasant or painful in patients, and it is sometimes impossible to be performed in children who do not tolerate a painful needle. In contrast, sEMG is less invasive and surveys a larger and therefore more representative area of the muscle. However, the necessary post-processing, the strongly varying signal-to-noise ratio, and, if used, the attachment of multiple electrodes prevented sEMG from becoming a diagnostic modality for neuromuscular patients in everyday clinical routines (Hogrel, 2005; Stålberg et al., 2019).

### Optically pumped magnetometer – A new tool for clinical diagnostics?

An elegant alternative to EMG is magnetomyography (MMG), which uses optically pumped magnetometers (OPM). MMG measures the magnetic fields generated by the electrical current flowing in the muscles. As the magnetic companion of EMG, it does not require skin contact as a source of potential noise, is contactless and, compared to nEMG, is therefore completely painless. Although MMG was already described by Cohen and Gliver in 1972, the field of the MMG was not pursued due to the technical limitations of the conventional superconducting quantum interference device (SQUID) magnetic sensors, which require cryogenic cooling to −269°C, are bulky and lack spatial flexibility. OPMs, on the contrary, are small, do not require cryogenic cooling, and enable recording muscle activity in individual anatomical situations, which leads to a revival of the field of MMG (for a detailed technical description of OPM sensors see: Shah and Wakai, 2013; Boto et al., 2017; Tierney et al., 2019). Since MMG and EMG signals originate from the same ionic currents, they also share comparable temporal and spectral profiles (Cohen and Givler, 1972; Parker and Wikswo, 1997), making MMG comparable to sEMG data. Since the magnetic permeability of human tissue is almost the same as in empty space, and the contactless sensors do not touch the skin, the signal is less distorted by additional noise, which suggests the potential superiority of the MMG technique (Schenck, 1996; Huigen et al., 2002; De Luca et al., 2010). Additionally, this also means that the technique can measure the electrical activity of deeper sections of the muscle and is therefore more representative of actual muscle activity than sEMG. Indeed, to date, MMG has shown a broad variety of applications for physiological muscle activity (Barchiesi et al., 2020; Llinás et al., 2020; Broser et al., 2021a,b; Marquetand et al., 2021), but the main focus of these studies was on physiological muscle activity, not on pathological muscle activity. A frequent critique of new technologies, especially in the field of biomagnetism, is that they fail to progress from scientific application to clinical routine. For example, magnetoencephalography (MEG) has led to many relevant findings in the field of neuroscience, but due to high expenses and time-consuming applications, it is not used in clinical routines on a daily basis. Here, both the low-cost of OPMs in the re-emerging field of MMG, and the option of performing muscle recordings outside a magnetically shielded chamber at the patients’ bedside using only portable shields for the limbs could lead to a solution to these shortcomings. To demonstrate for the first time that OPM-MMG is capable of being a new modality for clinical application, we performed a proof-of-principle study on three patients with neuromuscular disease performing an isometric contraction of the left rectus femoris muscle. Here, we compared the timeline of the signals qualitatively, as usually done in daily clinical routines, and also used quantitative parameters such as the power spectrum density (PSD) and the signal standard deviation (SD) to analyze muscle activity.

## Materials and methods

### Participants

Three patients with different neuromuscular diseases, such as Myotonica Congenita (MC), Transthyretin Amyloidosis in combination with Pompe’s disease (ATTR), and Charcot-Marie-Tooth disease, type 2 (CMT2), were included in our study (for detailed patient characteristics, see Table 1). Patient ATTR also suffered from Pompe’s disease, combining neurogenic and myogenic muscle damage, which was clinically and electrophysiologically less pronounced than aspects of ATTR. Therefore, patients ATTR and CMT2 represented neurogenic muscle damage (ATTR = severe, CMT2 = mild), which was diagnosed by an experienced clinician based on clinical appearance and available clinical nEMG-recordings. The patient MC served as a quasi-non-neurogenic control patient, with no clinical or electrophysiological signs of neurogenic muscle damage. Furthermore, seven healthy participants (four female, three male, mean age: 26.3 ± 2.9 years) served as healthy controls (HC). Participants from the control group come from a previously published study (Sometti et al., 2021). The study was approved by the ethics committee of the University of Tübingen (project number 692/2020B01), meeting the demands of the World Medical Association. All participants provided written consent.

**TABLE 1:** Patient characteristics.

ID	Gender	Age	Disease	Medical research council score of the rectus femoris muscle force
1	M	28	Myotonia congenita (MC)	5/5
2	M	58	Transthyretin-amyloidosis (ATTR) and Pompe’s disease	3/5
3	M	55	Charcot-Marie-Tooth disease, Type 2 (CMT 2)	5/5

The medical research council score (MRCS) of the rectus femoris muscle force ranges from 0/5 (no muscle contraction and muscle action) to 5/5 (normal force).

### Experimental setup

**FIGURE 1:**
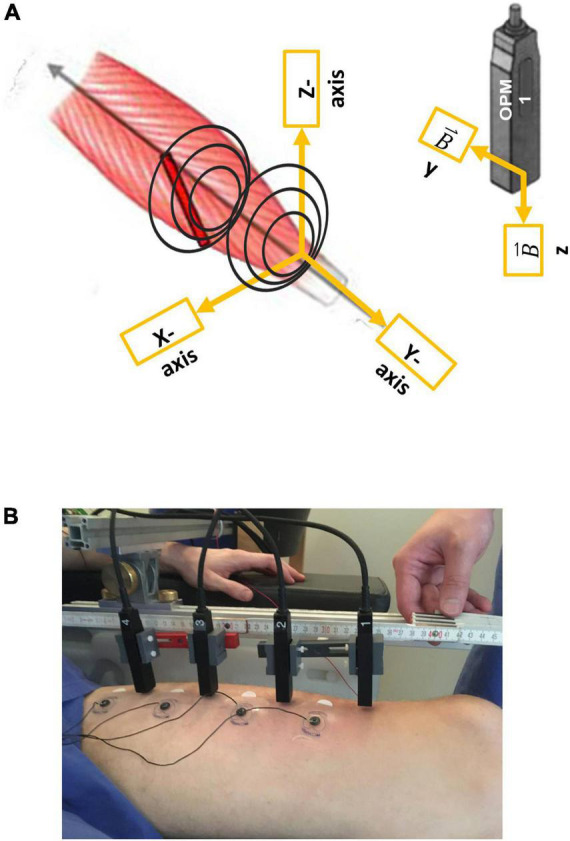
Illustration of the vectorial components of optically pumped magnetometers (OPMs), respectively, to the rectus femoris muscle **(A)** and the measurement setup **(B)**. Note that OPM 1–4 were placed from the distal (OPM 1) to the proximal (OPM 4). In addition, surface electrodes (sEMG 1–4) were placed in between the OPMs.

The study was conducted at the MEG Center of the University of Tübingen (Germany) and took place inside a magnetically shielded room (Ak3b, VAC Vacuumschmelze, Hanau, Germany). All participants were placed in a comfortable chair, extending their legs on the chair at a controlled knee angle of 150°. During the experiment, a counterpart would press down the left ankle, and the participants were asked to continuously push back with maximum force possible. Participants performed a total of three isometric contractions of the left rectus femoris muscle with a duration of 60 s and a pause of 30 s in between each block (Figure 1A) (For a more detailed description of the experimental setup, see Sometti et al., 2021). In patients, an isometric contraction with a duration of 60 s was not possible. Therefore, the patients were instructed to push with maximum force for as long as possible.

### Magnetic and electrophysiological recordings

Prior to the measurement, an ultrasound (Mindray TE7, 14 MHz-linear probe) was conducted by a qualified examiner (JM) to determine the longitudinal axis of the left rectus femoris muscle. Four optically pumped magnetometer (OPM) (QZFM-gen-1.5, QuSpin Inc., Louisville, CO, United States) were placed in line from distal to proximal along the longitudinal axis of the muscle, with a distance of 6 cm between each sensor. The first sensor was placed 6 cm proximal to the patella, and within 1–3 cm (mean distance 2.03 ± 0.65 SD cm) to the skin. The OPMs were held with a custom-built plastic frame attached to an aluminum structure. Utilized OPMs could record only two orthogonal components of the magnetic field vector at a time, Y and Z, respectively, the Y-axis being aligned to the longitudinal axis of the left rectus femoris muscle, and Z representing the orthogonally perpendicular component of the magnetic field (for OPM sensor coordinates, see Figure 1A). In line with the OPMs, four paramagnetic surface electrodes (Conmed, Cleartrace2 MR-ECG-electrodes) were placed between the OPM sensors with an inter-electrode distance of 6 cm to simultaneously record electromyographic signals (Figure 1B). The ground electrode was placed on the right shoulder, the reference electrode was placed on the lateral epicondyle of the right femur. To the best of our knowledge, no paramagnetic disposable EMG electrodes are commercially available; therefore, we shielded MR-ECG electrodes by twisting a paramagnetic cable wire around the electrode button, as previously done by Marquetand et al. (2021).

### Data acquisition

The OPM and sEMG data were simultaneously recorded using the MEG system (CTF Omega 275, Coquitlam, BC, Canada) and its included EEG channels for EMG data. Both the OPM and sEMG data were sampled at 2,343.8 Hz. The utilized OPMs provide a magnetic field sensitivity of 15 fT/Hz and a bandwidth of 3–135 Hz, an operating range below 200 nT, and a dynamic range of a few nT. To adapt to a non-zero magnetic background field, the sensors are equipped with internal compensation coils (Osborne et al., 2018) to cancel magnetic background fields of up to 200 nT (operating range). For all measurements, an internal output gain factor of 3 was applied in the user interface of the sensors, which corresponds to a conversion factor for the analog output of the OPMs to a magnetic flux of 1.11 nT/V.

### Interference pattern and visual inspection of the signal timeline

During maximum contraction, single muscle fibers are firing at different frequencies and amplitudes, which overlap. Electrophysiologically, the interference patterns of many muscle fibers and many different frequencies can be recorded. The interference was qualitatively evaluated by experienced clinicians (JM and MSB), referring to density and amplitude. In healthy individuals, this interference pattern is dense, and amplitudes are of similar sizes; in neurogenic patients, the interference pattern is expected to be dispersed and to have high amplitudes (Mills, 2005).

### Preprocessing and data analysis

Data preprocessing was performed using Matlab R2019b (The MathWorks, Natick, MA, USA). In addition, the FieldTrip toolbox (Oostenveld et al., 2011) was used. In order to explore the signal properties of simultaneous OPM and EMG recordings, we focused on a single channel located at the muscle belly, and therefore chose corresponding EMG 2 and OPM 2 (Figure 1B). After demeaning, both EMG and OPM datasets were filtered with a 10 Hz high-pass, zero-phase, sixth-order Butterworth infinite impulse response (IIR) filter. To remove powerline noise and instrumentation artifacts, a band-stop, zero-phase, fourth-order IIR filter was applied (frequency ranges in Hz: 48–52, 98–102, 148–152, 161–163, 195–202, and 248–252).

For both healthy controls and patients, periods of contraction in the sEMG and OPM recordings were manually selected. Then, these data segments were divided into 3 s non-overlapping windows. To characterize the signal properties in each of these windows, two parameters in the time-frequency domain were calculated. After calculation of our parameters, outliers were removed using the function “rmoutlier” in Matlab. These are the power spectral density (PSD) in the interval 20–100 Hz, where we consider most of the muscle activity to take place, and the signal standard deviation (SD) as a proxy for signal variability. For the calculation of the discrete Fourier transform, we used the “fft” algorithm implemented in Matlab. Altogether, we extracted *n* = 309 windows for HC, *n* = 8 windows for MC, *n* = 13 windows for ATTR, and *n* = 11 windows for CMT2.

### Statistics

The low number of windows extracted from our patients prevented us from verifying whether our extracted parameters were normally distributed. Therefore, we performed the Kruskal–Wallis test. We compared PSD and SD between groups (HC, MC, ATTR, and CMT2) and within sensors (EMG, OPM Y, and OPM Z). This resulted in a total of six tests. The tests were considered statistically significant if *p* ≤ 0.05. In the case of statistical significance, a multiple comparison test was performed to identify differences between the groups using the “multcompare” function in Matlab. Bonferroni correction for multiple comparisons for *n* = 6 different contrasts was applied setting the significance level to *p* ≤ 0.008.

## Results

In total, we analyzed a set of 1,260 s (180 s in each of the seven subjects) for HC, 38.23 s for MC, 52.35 s for ATTR, and 55.98 s for CMT2.

### Reduced interference and high amplitudes in patients with neurogenic muscle damage

**FIGURE 2:**
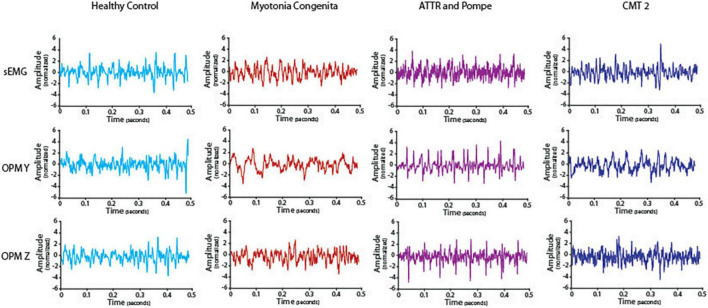
Timeline of the electromyography (EMG) and optically pumped magnetometer (OPM) signals in one healthy control and all three patients. Depicted is a representative episode of 0.5 s during maximum contraction after preprocessing. ATTR showed reduced interference and high amplitudes, especially in OPM Y but also in OPM Z compared to HC. EMG data from ATTR were still very noisy after preprocessing.

We visually compared the timeline of the signal of the sEMG and OPM-MMG between the HC and the three neuromuscular patients. Differences were most pronounced in the severely affected ATTR, especially in OPM Y, and to a smaller extent in OPM Z (see Figure 2). The interference pattern was dispersed during maximum contraction, and the amplitudes were higher compared to HC. MC and CMT2 did not show distinct differences compared to HC.

### Reduced power spectrum density and signal variability

**FIGURE 3:**
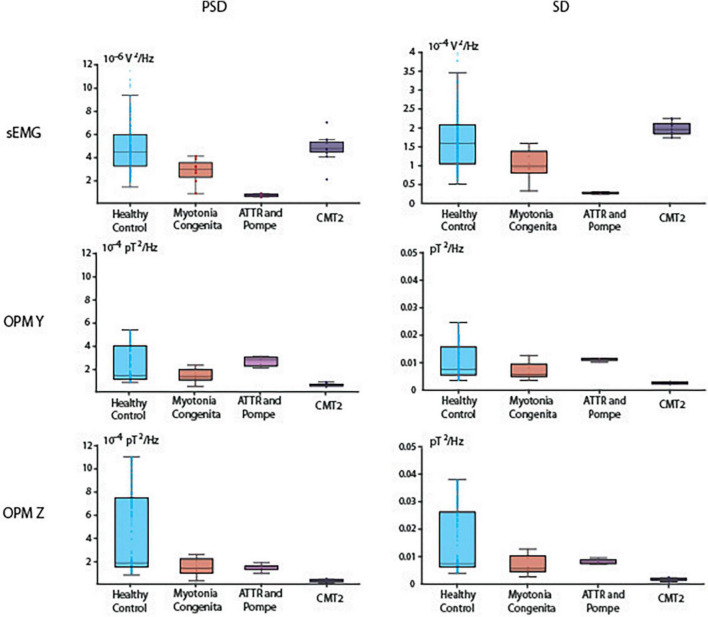
Boxplots of power spectral density (PSD) in the interval of 20–100 Hz and the signal standard deviation (SD). Depicted are median, and the 25th and 75th percentiles.

First, we performed a general analysis, which revealed that PSD was different between our groups in the EMG [H(3) = 47.28, *p* < 0.001], OPM Y [H(3) = 32.23, *p* < 0.001], and OPM Z [H(3) = 52.39, *p* < 0.001] recordings (Figure 3). SD was also different between our groups in the EMG [H(3) = 46.40, *p* < 0.001], OPM Y [H(3) = 32.98, *p* < 0.001], and OPM Z [H(3) = 37.55, *p* < 0.001]. See Figure 3 for more details.

Reduced PSD was detected in both patients with neurogenic muscle damage (ATTR and CMT2) compared to HC. Especially in ATTR, this correlates with reduced muscle mass due to progressive atrophy. PSD in the EMG was higher in HC compared to ATTR (*p* < 0.001). PSD in the OPM Y was higher in HC compared to CMT2 (*p* < 0.001), and PSD in the OPM Z was higher in HC compared to ATTR (*p* < 0.001) and to CMT2 (*p* < 0.001). For descriptive information regarding the median and interquartile range (IQR) of PSD, see Supplementary Table 1. The patient suffering from MC did not show distinct differences in comparison to HC, which is reflected by the widely overlapping box plots in OPM Y and OPM Z (Figure 3).

In OPM-MMG, both patients with neuropathic muscle damage (ATTR and CMT2) showed a marked reduction in the variability of the power spectra, indicated by the very narrow SD. In the EMG, SD was higher in HC compared to ATTR (*p* < 0.001). In the OPM Y, SD was higher in HC compared to CMT2 (*p* < 0.001), and higher in ATTR compared to CMT2 (*p* < 0.001). The same patterns were observed in the OPM Z. For detailed information regarding the IQR of SD, see Supplementary Table 2. Again, the patient suffering from MC did not show distinct differences in OPM Y and OMP Z in comparison to HC.

## Discussion

This proof-of-principle study is, to the best of our knowledge, the first study to show that altered voluntary muscle activity due to neurogenic damage of skeletal muscles is detectable using non-invasive OPM, which paves the way to perform further and large-scale studies in neuromuscular patients. It is clinically relevant that the OPM-MMG signal is visually comparable to sEMG, showing a dispersed interference pattern with a tendentially higher signal amplitude (Figure 2) and reduced variability of the frequency spectrum (Figure 3) at maximal voluntary contraction, as expected in neurogenic muscle damage due to motor neuron loss.

### Optically pumped magnetometer-magnetomyography as a potential clinical diagnostic

In the clinical electrophysiological routine, the interference pattern is mainly evaluated qualitatively, i.e., only visually based on the EMG curve, using nEMG during the maximum contraction of a muscle. When the interference pattern is dispersed and shows high amplitudes at the same time, this indicates a loss of motor neurons, reflecting the compensatory activity of the remaining motor units (Hansen and Ballantyne, 1978). This purely visual analysis was also possible in the OPM-MMG and showed corresponding findings in our patients (Figure 2). The patient with the most severe neurogenic muscle damage (ATTR, paretic muscle) showed dispersed interference alongside high amplitudes, which was not observed in HC. This finding is typical of neurogenic muscle damage (Dumitru et al., 2002; Mills, 2005; Preston and Shapiro, 2012). However, the patient with less neurogenic muscle damage (CMT2) did not clearly show this typically dispersed interference pattern along with high amplitudes, which might be because this patient still had enough motor units, as he was clinically unaffected (i.e., no paresis of the measured muscle). Considering the findings in the patient with severe neurogenic muscle damage (ATTR), OPM-MMG might offer the possibility of a non-invasive method for a pure qualitative, i.e., visual, analysis of the muscle signal, but this needs to be further validated in large-scale studies.

In addition to this purely visual-qualitative analysis of the signal time course, we introduced two new quantitative parameters: PSD and SD. In the case of damage, the variability of the signal should decrease at maximum voluntary activity. In fact, a muscle has essentially two possibilities for generating and maintaining force over time: first by recruiting more motor units and second by increasing the frequency of its innervation (Adrian and Bronk, 1929; Seyffarth, 1940; De Luca and Hostage, 2010). In the case of motor neuron loss, fewer motor units are available; that is, the possibility of recruiting more motor units and thus different discharge frequencies is no longer possible, which leads to a decrease in the variability of the signal, indicated by the narrow SD and the small IQR (Figure 3 and Supplementary Table 2). This finding could be demonstrated in both patients with neurogenic muscle damage (ATTR and CMT2), which also underlines the possibility of using OPM-MMG to demonstrate typical changes resulting from neuropathy. Since these changes were already visible in the only mildly affected patient CMT2, it might be a more sensitive marker for detecting early neurogenic remodeling. Spectral analysis algorithms might be easily established in clinical routines and could serve as objective parameters to quantify muscle damage.

The pure electrophysiological features (dispersed interference, higher amplitudes, and low frequency variability) of neurogenic muscle damage can already be visualized by a simple one-channel sEMG (Stålberg et al., 2019), giving information on the active number of motor units and, based on the larger area included, being even more representative compared to nEMG. Based on the sEMG signal data, we hypothesized that it should also be possible to detect electrophysiological changes in the (preprocessed) OPM-MMG signal, which was also the case for the interference pattern. However, counterintuitively to our expectations, in the quantitative analysis (see “Results” and “Reduced power spectrum density and signal variability”, Figure 3) of the EMG and OPM-MMG signals, we found that they were visually not fully concordant (PSD and SD in ATTR being lower compared to HC in sEMG, but not in OPM, and also PSD and SD in CMT 2 being lower compared to HC in OPM, but not in EMG). The reason for this must remain unanswered for now, given the small number of cases, but motivates us to conduct larger studies.

To gain information about the single motor unit, more complex methods, i.e., high-density sEMG (Hogrel, 2005), are needed, being both more expensive and quite time consuming due to the high effort in their application. Additionally, the more complex the setup and the more electrodes needed, the higher the susceptibility to noise. In OPM, it should be possible to combine both aspects, gaining information on a large and representative area and an analysis of the single motor unit by implementing new parameters and standardized analysis algorithms. It should be possible to use only one setup and one single measurement to provide multiple information on different electrophysiological aspects without a time-consuming setup or multiple measurement - both indispensable in clinical routine.

Furthermore, as stated above, OPM is not dependent upon tissue conduction and can measure the electrical activity of deeper sections of the muscle. Besides a more representative measurement of pure muscle activity, it is also helpful to detect deep muscle fasciculations, i.e., ALS, and also might potentially be used for a more accurate motor unit number estimation (MUNE).

### Methodological considerations, limitations, and options

We hypothesized that OPM-MMG is a non-invasive method for detecting pathological changes in muscle activity in neuromuscular patients. Therefore, we designed this proof-of-principle study including only three patients, nevertheless paving the way for additional studies in a larger number of patients, including early-and late-state patients with neuromuscular muscle damage. We also wanted to address the aspect that, besides the necessity of large studies gaining standard values, finding a diagnosis is always an individual process dealing with data from only a single patient. Besides the option of replacing conventional nEMG with a non-invasive method, new, and objective parameters might also be developed to detect early and subtle changes in muscle activity.

The patient MC served as a quasi-non-neurogenic control patient, and we did not expect any of the typical changes in neurogenic muscle damage. Visual inspection of the interference pattern revealed a dense pattern with uniform amplitudes, and SD did not show significant differences between HC and MC. However, statistical analysis of the PSD revealed a significant difference between HC and MC in EMG, which was reduced in MC. The differences are not powerful and on the margin of significance but might be due to subtle weakness. Unfortunately, we did not integrate force measurements in this paradigm, an aspect to be addressed in future studies, and might even provide further information about the correlation between clinical and electrophysiological changes.

As previously elaborated, the MMG signal originates from the ionic current of multiple muscle fibers. However, muscle fibers are not always arranged in parallel, but, depending on the examined muscle, can also be fusiform, convergent, or even circular, affecting the signal and causing interference phenomena. Based on the anatomy of the examined muscle, a geometric source model might be established (work in progress), comparable to modeling methods being used in MEG/EEG, to delineate individual sources, i.e., single muscle fibers, contributing to the signal. Besides this aspect, the signal is also secondarily dependent on the distance between the source and the sensor. The pure contraction of the muscle alternates this distance and, due to the muscle belly, even causes differences between the individual sensors. A standardized measure of the distance between the muscle and source needs to be established.

The spectral bandwidth of physiological muscle activity ranged from approximately 20–300 Hz. Due to the physical properties of the sensors, sensitivity and bandwidth behave in an opposite way, with high sensitivity of the magnetic field narrowing the bandwidth, and with a broad measurable bandwidth reducing sensitivity. In our experiment, we decided to use OPM sensors combining a high sensitivity of the magnetic field (15 fT/Hz) and a bandwidth located in the lower frequency spectrum (3–135 Hz), where we expected most muscle activity to be located, however, limiting the detection of high frequency muscle activity. The balance between sensitivity and bandwidth is one of the main limitations of the application of magnetometers in MMG. Furthermore, OPM sensors are limited to recording only two orthogonal components of the magnetic field vector at a time (Y and Z, OPM sensor coordinates, Figure 1A), with the necessity of repeating the measurements after rotating the sensors by 90° around their z-axis to record all dimensions of the magnetic field. Further research to develop triaxial sensors is currently in progress and might overcome this disadvantage.

## Conclusion

In this proof-of-principle study, we introduced OPM-MMG as a non-invasive method to detect neurogenic muscle damage in patients. As we demonstrated, it is not only possible to adapt visual-qualitative analyses, but also to establish new parameters by using standardized analysis algorithms that offer quantitative evaluation. The small sensor size and the option of only small shielding devices needed for the limbs might help manage the transition from purely scientific to the clinical application of MMG.

## Data availability statement

The original contributions presented in this study are included in the article/Supplementary material, further inquiries can be directed to the corresponding author.

## Ethics statement

The studies involving human participants were reviewed and approved by Ethik-Kommission der Universität Tübingen. The patients/participants provided their written informed consent to participate in this study.

## Author contributions

LS conceptualized and conducted the analysis, prepared the figures, and drafted the manuscript. MSB prepared the figures, drafted the manuscript, and supervised the work. JM performed the recordings, drafted the manuscript, and supervised the work. TM provided and set up the OPMs and revised the manuscript. DS helped to conduct the study and revised the manuscript. SB, HC, AG, HL, PM, CK, CB, PB, and MS revised the manuscript. All authors read and approved the revised manuscript.

## Abbreviations

ATTR: transthyretin amyloidosis
CMT 2: charcot-marie-tooth disease, type 2
EMG: electromyography
EEG: electroencephalography
IQR: interquartile range
MC: myotonia congenital
MD: myotonic discharge
MEG: magnetoencephalography
MMG: magnetomyography
nEMG: needle electromyography
OPM: optically pumped magnetometer
PSD: power spectrum density
SD: signal standard deviation
sEMG: surface electromyography
SQUID: superconducting quantum interference device.
