# Supplementary material for: Optically pumped magnetometers detect altered maximal muscle activity in neuromuscular disease
Source: Front Neurosci. 2022 Nov 29;16:1010242. doi: 10.3389/fnins.2022.1010242 (PMC9745080; doi:10.3389/fnins.2022.1010242)
Supplement: Supplementary file 1 [file Data_Sheet_1.docx]

|  | EMG | OPM Y | OPM Z |
| --- | --- | --- | --- |
| HC | 4.5 ± 2.7*10^-6 V²/Hz | 1.4 ± 3.1*10^-4 pT²/Hz | 2.1 ± 6.5*10^-4 pT²/Hz |
| MC | 3.0 ± 1.2*10^-6 V²/Hz | 1.3 ± 1.0*10^-4 pT²/Hz | 1.6 ± 1.4*10^-4 pT²/Hz |
| ATTR | 0.8 ± 0.2*10^-6 V²/Hz | 2.9 ± 0.8*10^-4 pT²/Hz | 1.7 ± 0.3*10^-4 pT²/Hz |
| CMT2 | 4.8 ± 0.9*10^-6 V²/Hz | 0.6 ± 0.2*10^-4 pT²/Hz | 0.4 ± 0.2*10^-4 pT²/Hz |

***Supplementary Material Table 1***: PSD median ± IQR

|  | EMG | OPM Y | OPM Z |
| --- | --- | --- | --- |
| HC | 1.6 ± 1.0*10^-4 V²/Hz | 0.8 ± 1.0*10^-2 pT²/Hz | 0.7 ± 1.9*10^-2 pT²/Hz |
| MC | 1.0 ± 0.6*10^-4 V²/Hz | 0.6 ± 0.5*10^-2 pT²/Hz | 0.6 ± 0.5*10^-2 pT²/Hz |
| ATTR | 0.3 ± 0.0*10^-4 V²/Hz | 1.1 ± 0.1*10^-2 pT²/Hz | 0.8 ± 0.1*10^-2 pT²/Hz |
| CMT2 | 2.0 ± 0.3*10^-4 V²/Hz | 0.3 ± 0.1*10^-2 pT²/Hz | 0.2 ± 0.1*10^-2 pT²/Hz |

***Supplementary Material Table 2:*** SD median ± IQR
